# Antidepressant use and psychosis hospitalization in persons with schizophrenia

**DOI:** 10.1192/j.eurpsy.2023.581

**Published:** 2023-07-19

**Authors:** A. Puranen, M. Koponen, M. Lähteenvuo, A. Tanskanen, J. Tiihonen, H. Taipale

**Affiliations:** ^1^Department of Forensic Psychiatry, Niuvanniemi Hospital; ^2^School of Pharmacy, University of Eastern Finland, Kuopio, Finland; ^3^Faculty of Pharmacy and Pharmaceutical Sciences, Monash University, Parkville, Australia; ^4^Department of Clinical Neuroscience, Karolinska Institutet; ^5^Centre for Psychiatry Research, Stockholm City Council, Stockholm, Sweden

## Abstract

**Introduction:**

Antidepressants are often used by persons with schizophrenia. These medications are used for a variety of symptoms, such as negative or depressive ones. Effectiveness of antidepressant use in persons with schizophrenia has rarely been studied in the real-world setting.

**Objectives:**

The aim of this study was to investigate the risk of hospitalization due to psychosis related to antidepressant use in persons with schizophrenia.

**Methods:**

This cohort study utilized data combined from Finnish nationwide registers. The study cohort included all 61 889 persons treated in inpatient care due to schizophrenia (defined as International Classification of Diseases, ICD, version 10 codes F20-F25 during 1972–2014 in Finland). National Prescription register data was utilized to obtain drug purchase data, and modelled into drug use periods with PRE2DUP (From Prescriptions to Drug Use Periods) method, developed by our research group. The follow-up covered the years from 1996 to 2017. Antidepressants (Anatomic Therapeutic Chemical classification system, ATC code N06A) were categorized by mechanism of action (non-selective monoamine reuptake inhibitors, TCAs, ATC-codes N06AA, selective serotonin reuptake inhibitors, SSRIs, N06AB and serotonin-norepinephrine reuptake inhibitors, SNRIs, including venlafaxine, milnacipran and duloxetine), and also on drug-substance level. Main outcome was hospitalization due to psychosis (ICD-10 diagnoses F20-F29) as the main diagnosis. We used within-individual design to compare the risk of outcome between the time periods of antidepressant use and non-use within the same person to minimize selection bias. Stratified Cox regression analyses were utilized to calculate adjusted hazard ratios (aHR) with 95% confidence intervals (CIs). These analyses were then adjusted for sequential order of treatments, time since cohort entry, use of antipsychotics, mood stabilizers, benzodiazepines, and Z-drugs.

**Results:**

The mean age of the study cohort was 46.2 (SD 16.0) years at cohort entry, and 50.3% of were males. Altogether 49.3% (N=30 508) of the study cohort used antidepressants during the follow-up (median 14.8 years, IQR 7.5-22.0), with citalopram and mirtazapine being the most commonly used antidepressants. The risk of psychosis hospitalization was lower during antidepressant use as compared to non-use (aHR 0.93, 95% CI 0.92-0.95). Use of SSRIs was associated with similar risk (aHR 0.91, 95% CI 0.89-0.93), followed by SNRIs (aHR 0.92, 95% CI 0.88-0.97) and TCAs (aHR 0.93, 95% CI 0.89-0.98). Considering individual drug substances, lowest risk were obserwed with use of sertraline (aHR 0.87, 95% CI 0.83-0.91), fluoxetine (aHR 0.88, 95% CI 0.83-0.91) and citalopram (aHR 0.92, 95% CI 0.90-0.95).

**Conclusions:**

Use of antidepressants was associated with a 7% lowered risk of hospitalization due to psychosis, and AD subgroups did not differ in their real-world effectiveness.

**Disclosure of Interest:**

A. Puranen: None Declared, M. Koponen: None Declared, M. Lähteenvuo Shareolder of: Genomi Solutions ltd, Nursie Health ltd, Springflux ltd, Grant / Research support from: Finnish Medical Foundation, Emila Aaltonen Foundation, Speakers bureau of: Sunovion, Lundbeck, Otsuka Pharma, Orion Pharma, Recordati, Janssen, Janssen-Cilag, A. Tanskanen Grant / Research support from: Janssen-Cilag, Eli Lilly, , J. Tiihonen Grant / Research support from: Janssen-Cilag, Eli Lilly, Consultant of: HLS Therapeutics, Orion, and WebMed Global, Speakers bureau of: Eli Lilly, Evidera, Janssen-Cilag, Lundbeck, Mediuutiset, Otsuka, Sidera, and Sunovion, H. Taipale Grant / Research support from: Janssen-Cilag, Eli Lilly, Academy of Finland, Speakers bureau of: Janssen-Cilag, Otsuka

